# Silver Diamine Fluoride Renaissance in Paediatric Dentistry: A 24-Month Retrospective and Cross-Sectional Analysis

**DOI:** 10.3390/medicina60010016

**Published:** 2023-12-21

**Authors:** Ruba Abdulrahim, Christian H. Splieth, Mhd Said Mourad, Annina Vielhauer, Manasi R. Khole, Ruth M. Santamaría

**Affiliations:** 1 Department of Preventive and Pediatric Dentistry, University of Greifswald, 17475 Greifswald, Germany; ruba.abdulrahim@stud.uni-greifswald.de (R.A.); splieth@uni-greifswald.de (C.H.S.); mhd.mourad@uni-greifswald.de (M.S.M.); annina.vielhauer@uni-greifswald.de (A.V.); manasi.khole1@uni-greifswald.de (M.R.K.); 2 Department of Orthodontics, University of Greifswald, 17475 Greifswald, Germany

**Keywords:** dental caries, silver diamine fluoride, treatment outcome, paediatric dentistry, parents

## Abstract

*Background and Objectives:* Silver diamine fluoride (SDF) has been incorporated into the treatment of dental caries in children, mainly in countries with high caries prevalence. In Europe, however, SDF started to gain popularity during the COVID-19 pandemic. This study aimed to investigate the efficacy of SDF and to evaluate dentists’/parents’ acceptance of SDF use in paediatric patients treated in a German university setting. *Materials and Methods:* A retrospective analysis of all patients treated with SDF between 2017 and 2020 was carried out. Only teeth with no reported clinical/radiographic evidence of irreversible pulpal inflammation were included. The outcome measures were success, minor failures (caries progression, reversible pulpitis) and major failures (irreversible pulpitis, abscess). The treatment acceptance by dentists and the parents of SDF-treated children was cross-sectionally evaluated using questionnaires. Descriptive statistics and Kaplan–Meier survival analysis were performed. *Results:* A total of 93 patients (mean age 5.3 ± 2.9 years) with 455 treated teeth (418 primary/91.9%; 37 permanent/8.1%) were included and followed up for up to 24 months (19.9 ± 10.5 months). SDF was used for dental caries (98.2%) and hypersensitivity relief on MIH teeth (1.8%). Most teeth did not show any failure (total success 84.2%). A total of 5 teeth (1.1%) showed minor failures, and 67 teeth (14.7%) showed major failures (*p* = 0.001). Success/failure rates were not affected by patient compliance, gender, dentition, or operator (*p* > 0.05). In total, 30 questionnaires were collected from parents (mean age 36.8 ± 6.4 years). SDF was applied on anterior (*n* = 2/6.7%), posterior (*n* = 15/50%) and anterior/posterior teeth (*n* = 13/43.3%). At the 1-week follow-up, 80% of parents noticed black teeth discoloration. Treatment satisfaction was higher for posterior (95.2%) than for anterior teeth (36.4%; *p* < 0.001). In the 27 responses from clinicians, SDF was generally considered a viable option in paediatric dentistry (*n* = 23; 85%). *Conclusions*: SDF was found to be effective and well-accepted by parents and dentists for caries inactivation in a paediatric dentistry German university setting.

## 1. Introduction

Dental caries in children remain a global oral health issue to this day. Multiple risk factors have been associated with this issue, such as poor oral hygiene and dietary habits as well as altered oral bacterial flora. The increasing prevalence, especially in countries with low socio-economic status, present as a burden and challenge to its management. Hence, biological-based minimally invasive treatment strategies have been widely advocated in the recent literature [1]. First developed in the late 1960s, silver diamine fluoride (SDF) has been a research topic of interest, particularly in the last decade [2]. It has been thoroughly investigated and several systematic reviews have demonstrated its effectiveness in arresting caries in primary teeth, showing up to 91% success rates with biannual application [3,4,5]. This led the World Health Organisation to list it as an essential medicine for the treatment of carious lesions [6]. SDF has been largely researched in countries with high caries prevalence and regions with limited access to dental care [7,8,9].

The growing evidence on its effectiveness has led to its adoption worldwide including, but not limited to, in Australia, Brazil, Hong Kong, Japan, and the United States [10]. On the other hand, in Europe, treatment with SDF has been very limited due to the lack of national guidelines and its restricted off-label use. It has been proposed as an alternative option for young children presenting with high caries levels or low cooperation for invasive restorative therapy. In this regard, the European Organisation for Caries Research (ORCA), the European Federation of Conservative Dentistry (EFCD) as well as the German Association of Conservative Dentistry (DGZ) published, in a recent consensus statement, a recommendation of high-strength that SDF can be successfully used in the treatment of ECC [11]. SDF became further widespread during the COVID-19 pandemic, as it is considered as a non-invasive, non-aerosol-generating procedure [10,12].

Considering the lack of research on SDF use in Europe, this study aimed to investigate the clinical efficacy and acceptance by dentists and parents of paediatric patients treated with SDF application in Germany in a university dental setting.

## 2. Materials and Methods

### 2.1. Ethical Approval and Study Outline

Ethical approval was obtained from the Research Ethics Committee of the University of Greifswald under protocol number BB-142/20. This study comprised two different designs: a retrospective analysis and a cross-sectional questionnaire-based analysis.

### 2.2. Retrospective Analysis

Electronic records of all patients treated with SDF at the Preventive and Paediatric Dentistry Department in the University of Greifswald, Germany were retrospectively collected and followed up to evaluate the effectiveness of SDF treatment.

Study sample and inclusion and exclusion criteria

All patients, irrespective of age or health status, without baseline clinical or radiographic signs/symptoms of pulpal/periapical pathology, who were treated with SDF at the University of Greifswald Paediatric Department between January 2017 and February 2020, and who had attended at least one follow-up appointment (until February 2022), were included in the study. Patient records with insufficient documentation were excluded.

Clinical procedure

The SDF product used was Riva Star^®^ (SDI Limited, Victoria 3153, Australia), which includes two bottles that are applied sequentially: bottle/step 1 corresponds to 38% SDF solution and bottle/step 2 contains potassium iodide (KI) solution (to lower the risk of black staining). The tooth is first cleaned, and debris removed. Soft tissues are protected using cotton rolls and petroleum jelly. SDF solution is then applied on the carious lesion using a micro brush after drying the tooth. The treatment was performed in a chairside setting by 8 dentists, 5 of whom were paediatric dental specialists and 3 were postgraduate paediatric dentistry (PD) students with at least 2 years of working experience, all following the department’s standard protocol for caries management in children and trained in treating children with SDF.

Outcomes

Recorded data comprised demographics (age, gender, address, medical status) and clinical baseline findings (d_3_mft/s-D_3_MFT/S index, clinical diagnosis, severity level of carious lesions using ICDAS index, radiographic and pulpal status, etc.). Outcome measures were assessed according to the last follow-up session using criteria modified from Innes et al. (2006) (Table 1) [13]. Data collection was based on the documentation obtained from the dental records. Documentation following treatment at the Preventive and Paediatric Dentistry Department was completed in a thorough and standardised manner using pre-set text for each procedure with the possibility to add or change notes when needed. Only patients with the sufficient documentation necessary for the study were included. Similarly, data were collected and decoded by the main investigator (RA) and reviewed by at least one co-investigator each time in a standardised manner using a Microsoft Excel (2020) spreadsheet prepared for the purpose of this study with the above-mentioned variables.

### 2.3. Cross-Sectional Questionnaire-Based Analysis

Questionnaires were created to assess dentists’ and parents’ acceptance of SDF treatment in children. The created questionnaires were first pilot tested with two paediatric dentists and five parents to ensure that they were comprehensible and acceptable to the target groups. No major flaws in the design of the questionnaires were revealed and the participating parents did not report any difficulty in answering the questions. Minor changes regarding the questionnaire structure, language, and format were made.

Sample size calculation

For the dentists’ questionnaire, all identified dentists using SDF at the University of Greifswald Paediatric Department were invited to participate in the study. However, for the parents’ questionnaire, the sample size was calculated according to initial results of the retrospective analysis (see Section 3.1). By assuming that around 400 patients received SDF annually, the sample size was calculated with population size = 400, confidence level 95% and a margin of error 20%, which, in turn, resulted in at least 23 participants to be included. Adding loss of responses due to missing data in the questionnaires (about 30%), a total sample size of 30 parents was determined.

Study participants
Parents/caregivers

The main investigator (RA) screened regular clinic attendees for eligible patients and consecutively recruited 30 participants according to the inclusion criteria. Only parents whose children were to be treated with SDF and who fully completed the first and follow-up questionnaires were included.

After obtaining a signed informed consent form, two questionnaires were distributed to the parents before and after one week of SDF application. The first questionnaire included a 12-item fill-in and Likert-scale questions covering participants’ demographics and assessing parents’ perception of the SDF procedure performed in terms of child comfort and behaviour during application and treatment duration. Meanwhile, the follow-up questionnaire consisted of 14-item fill-in and Likert-scale questions. The primary survey question was whether dark staining on the treated teeth was noticed by the parents or not, and if noticed, their opinion about it. Possible responses ranged from “very acceptable” to “very unacceptable”. The secondary questions further explored parents’ opinion of 3 main aspects related to the treatment: aesthetics, pain/complaints and SDF as a treatment option of carious lesions.

Dental practitioners

The dentists surveyed included paediatric dental specialists and post-graduate PD students all treating children on a regular basis. The questionnaire included 4 fill-in items regarding dentists’ experience and qualifications and 12 Likert-scale statements aimed at exploring dentists’ acceptance and experience with SDF. Possible responses ranged from “totally agree” to “totally disagree”. To explore acceptance, dentists were asked if they considered SDF a good treatment option for ECC or avoided it due to the associated black staining.

### 2.4. Statistical Analysis

Statistical analysis was carried out using SPSS (IBM Corp. Released 2017. IBM SPSS Statistics for Mac, Version 25.0. Armonk, NY, USA: IBM Corp.). Descriptive statistics were applied to describe patient characteristics and conditions of treated teeth. Chi-square statistics were used to test relationships between categorical variables. Survival analysis using the Mantel–Cox method and a Kaplan–Meier curve were used to report mean time until treatment failure as well as the Log-rank test. A Mann–Whitney *U* test was performed to test the differences in the acceptance of SDF treatment. The level of significance was set at 0.05.

## 3. Results

### 3.1. Patient Profiles and Characteristics

The dental records of 1202 patients were initially retrieved. The data were filtered and assessed according to the inclusion criteria. In total, 93 patients had sufficient documentation and attended at least one follow-up appointment. The demographic and clinical characteristics are presented in Table 2.

The patients’ age ranged from 1 to 17 years (±5.3 years). A sum of 455 teeth were treated with an average of 4.9 teeth per patient. Most of the treated teeth were primary teeth (*n* = 418; 92%). Only 41 patients (44%) had a recent radiograph available within the last year, almost all (93%) showing carious lesions at the dentin level (ICDAS 4–6). A total of 52% of the patients had a very negative or negative cooperative level. Dental caries was the main reported diagnosis (98.2%), along with a few cases of hypersensitivity due to MIH (*n* = 8; 1.8%). Patients mean d_3_mft/D_3_MFT was 6.3/2.5. The patients were treated by a postgraduate paediatric dentistry student (46.2%) or by a paediatric dental specialist (53.8%). Regarding the side effects of SDF application, only a few cases of sensitivity (7.5%) were reported during the application of the product. Other than the black staining of the carious lesions (98%), no adverse effects were reported in this study.

### 3.2. Clinical Efficacy and Indication

After 2 years of SDF application (19.9; SD = 10.5 months), follow-up data were collected, and the outcome of the treatment was evaluated according to the success/failure criteria (Table 3). The SDF treatment showed an overall success rate of 84.2%. There were 5 teeth (1.1%) presenting with minor failures and 67 teeth (14.7%) with major failures (*p* = 0.001). Besides dental caries, SDF was also used in the treatment of MIH-associated hypersensitivity in permanent first molars. Out of the eight treated permanent first molars, five required further restorative treatment due to persisted hypersensitivity, and one was extracted under general anaesthesia. Excluding MIH, the success rate of treated teeth due to dental caries was 85.2% with around two-thirds (57.2%) of carious teeth arrested, but not restored. The other 42.8% of teeth were restored after lesion inactivation.

**Figure 1:**
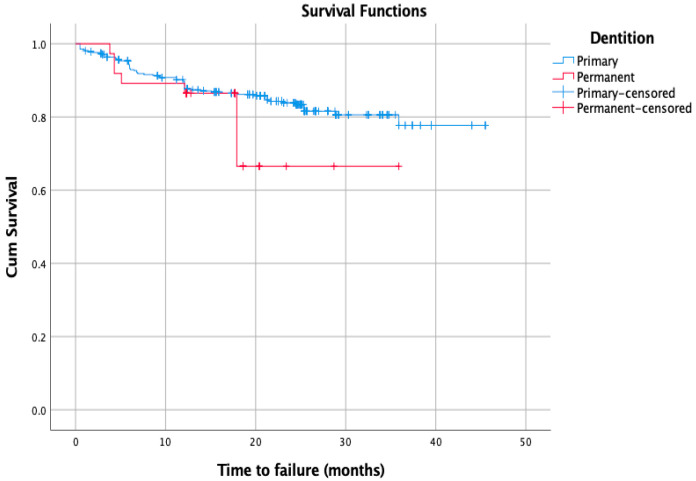
Kaplan–Meier survival curve showing survival time of SDF-treated teeth until failure.

After SDF application, the mean survival time for primary and permanent teeth was 38.8 months (95% confidence interval 37.2 to 40.3) and 28.3 months (95% confidence interval 23.7 to 32.8), respectively (Figure 1). The log-rank test was run to compare the primary and permanent dentition in terms of time until failure. No significant difference between both groups was found (*p* = 0.19). The results of the study were not statistically significant when success/failure rates were compared according to patient compliance, gender, dentition, or medical condition (*p* > 0.05).

### 3.3. Parents’ Acceptance and Satisfaction

Initially, 42 responses were collected. In total, 30 participants fully completed the follow-up questionnaire and were included in the study. The participant parent was mainly the mother of the child (*n* = 27; 90%) with a mean age of 37 years (±6.4 SD). SDF treatment was used on anterior teeth in 2 children (6.7%), posterior teeth in 15 children (50%), and both anterior and posterior in 13 children (43.3%). The parents had varying educational backgrounds from secondary school (5%) up to postgraduate studies (19%), with around 46% of parents having completed an apprenticeship, i.e., a vocational training programme. Among other reasons like regular check-up (27%) and pain (10%), the main reason behind the dental visit was dental caries in 63% of the participants.

**Figure 2:**
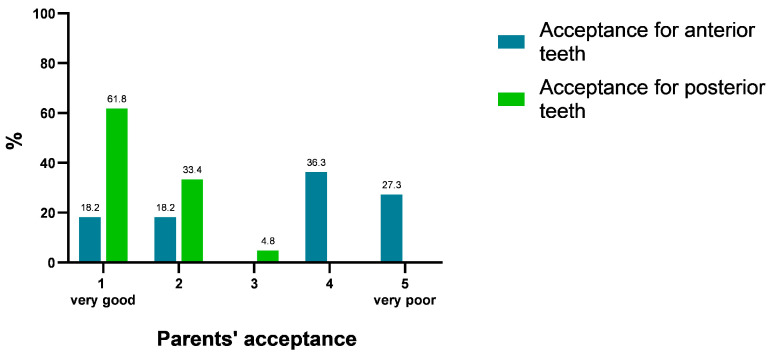
Parents’ acceptance of dark staining associated with SDF treatment. Likert Scale (1–5) from very good to very poor acceptance of dark discoloration.

At the first visit, there was an overall acceptance (70%) of the procedure in terms of child comfort. At the follow-up, 80% of the parents noticed dark staining on the treated teeth but around 71% assessed the treatment received as very acceptable or acceptable. However, satisfaction with treatment was higher for posterior teeth (95.2%) than for anterior teeth (36.4%; *p* < 0.001). When asked about the need for an aesthetic restoration, 43.3% responded with ‘agree’. Around 93% responded with ‘disagree’ when asked if they regret receiving the treatment due to its discoloration and 97% chose ‘agree’ for using SDF to arrest caries. Overall, there was high acceptance of SDF treatment of caries among the participating parents (Figure 2).

### 3.4. Dentists’ Experience and Knowledge

In total, 27 responses were obtained. Among these, 56% (*n* = 15) were paediatric specialists, 11% (*n* = 3) dental practitioners, and 33% (*n* = 9) post-graduate students, all treating paediatric patients on a regular basis.

All participants in this study, expect one, confirmed their awareness of the SDF protocol. Around 60% (*n* = 16) of the respondents agreed that they confidently use SDF based on the researched evidence for its effectiveness. The importance of obtaining a consent form from the parents was also confirmed by all participants.

Most of the participants (*n* = 23; 85%) consider the use of SDF as a good treatment option for ECC, and 67% (*n* = 18) would prefer it over other treatment options for children with ECC, as well as for the treatment of carious lesions in anxious/low cooperative children (*n* = 22; 81%). All participants, except one (96%) who “somewhat disagrees”, believe that SDF can be considered as an interim procedure to arrest carious lesions and win time until a restorative therapy can be delivered or until the child can be treated under general anaesthesia. Table 4 shows a summary of the main findings obtained from the questionnaire.

## 4. Discussion

This study explored the use of SDF in terms of clinical efficacy (2-year follow-up) and acceptance by the dentists and parents of paediatric patients treated in Germany in a specialised university dental setting. SDF was primarily used to arrest active carious lesions in young patients (mean age = 5.3 years) presenting with high caries risk and experience (d_3_mft/D_3_MFT = 6.3/2.5) and low cooperation levels.

Regarding the caries experience of treated patients (d_3_mft/D_3_MFT of 6.3/2.5), these figures are particularly high in comparison to average values for children in primary and permanent dentition in Germany. For 3-year-olds, a weighted mean d_3_mft value of 0.48 has been reported [14]. However, in the present study, this group presented almost 13 times higher d_3_mft levels than the average value for the whole population. In addition, for the whole sample the “d_3_/D_3_” component of the d_3_mft/D_3_MFT index corresponded to 78% in primary teeth and 65% in permanent teeth of the whole caries experience. Considering the high treatment need of the whole sample, as well as the limited ability to cooperate with dental procedures at a young age, without SDF, the only likely treatment option would have been treatment under dental general anaesthesia (DGA). Although the clinical success of dental procedures under DGA is considered very high in the medium-term, this procedure increases the health risk for young patients as well as the economic burden for public health systems [15,16,17].

The selected caries management modality in this study followed recent guidelines and evidence-based recommendations on the use of non/minimally invasive treatment modalities like SDF [18,19]. In total, 455 teeth were treated, most of which were primary teeth (92%). An overall success rate of 84.2% of SDF-treated teeth after 2-year follow-up was observed. Excluding MIH teeth, the success rate of treated teeth increases to 85.2%. Several systematic reviews and an umbrella review have reported caries arrest rates ranging from 51% to 91% in primary teeth [20,21,22,23]. The studies included assessed the effectiveness of SDF in arresting carious lesions in children in comparison to a placebo, fluoride varnish, or the use of atraumatic restorative treatment, including anterior and posterior teeth, annual or biannual application and with varying concentrations of SDF. This explains the wide range reported according to each study and its setting. However, all systematic reviews agree that there is strong evidence for the efficacy of SDF in arresting carious lesions in children [24]. Similarly, the present study reported that 57.2% of the successfully SDF-treated carious teeth did not require any further treatment/restoration. The rest (42.8%) were further restored at subsequent visits mostly with GIC (SMART-Technique) or the Hall technique (SMART-Hall).

On the other hand, carious teeth categorised under failure were teeth that developed pathological signs/symptoms of irreversible pulp deterioration. Many of these teeth had to be treated with pulpotomy/extraction under DGA. The mean time after SDF application until DGA treatment was almost 1 year (10.7 months). So, even in these cases, SDF may be considered beneficial to buy time until an appointment for DGA could be arranged or to reduce the implied risks of DGA at young ages [17].

In this study, SDF was mainly used for the treatment of carious lesions; however, attempts to reduce dental hypersensitivity using SDF were also seen in a few cases of MIH. Since SDF is a dentin-desensitizing agent, its use in reducing dentin hypersensitivity associated with MIH is not novel [25]. The number of MIH-treated teeth in this study was small (*n* = 8) and most teeth required further restorative treatment to reduce hypersensitivity. In addition, due to the per se limitations of retrospective analyses, the severity of hypersensitivity and the degree of MIH could not be accurately reported in this study. It is therefore difficult to draw conclusions on the effectiveness of SDF in MIH-diagnosed teeth. However, one clinical trial has recently reported significant reductions in hypersensitivity following the application of SDF and SMART sealants on MIH teeth for up to one year [26].

Other than the dark staining of the carious lesions, no adverse effects were reported. In seven patients (7.5%), sensitivity upon application of the SDF solution was reported. Most of these patients were under 5 years of age and with a very negative or negative level of cooperation. This could explain the difficulty faced by the child receiving the treatment and perceiving it negatively. While SDF is biocompatible and safe to use [21], its direct application to vital pulp has been shown to cause pulpal necrosis [27]. Therefore, correct diagnosis is imperative prior to treatment with SDF in deep carious lesions.

A high response rate of 93% was obtained from the questionnaire distributed to the dentists who treated the patients in this study. Most participants considered SDF a viable treatment option for ECC, especially for young children presenting with dental anxiety and low compliance, since it is a simple procedure. Dental professionals in the UK have also reported similar views [28]. Also, almost all participants used SDF to buy time prior to restorative therapy or treatment under DGA. This opinion was also reported in a survey of paediatric dentists in the US [29]. Similar minimally invasive procedures for the treatment of caries in children, such as the Hall technique using preformed metal crowns and non-restorative caries control, were also found to be easy procedures for dentists and favoured by the children [30].

However, the dark staining side effect associated with SDF was reported by many practitioners as the main barrier to its use [28,31,32,33]. In this study, few dentists reported parents rejecting this treatment option due to aesthetic concerns. It was, therefore, crucial to explore parents’ acceptance and satisfaction with SDF, since this product has been recently introduced and is not the most common treatment modality in Germany. Parents of treated children were surveyed prior to and after the application of SDF. Overall, parents showed a high acceptance of the procedure in terms of treatment time and child comfort and the majority assessed the treatment received as satisfactory with higher acceptance rates for posterior than for anterior teeth. A similar pattern was found in other studies that interviewed parents in the U.S. regarding their opinion of the discoloration following treatment with SDF [34,35], whereas a study from Saudi Arabia showed high disapproval rates, although the parents were interviewed and presented with photos and scenarios without the actual use of SDF [36]. It is important to note that the perception of aesthetics and beauty is subjective and influenced by several factors such as age, level of education and culture [37]. Child-related factors, such as the location of the tooth and cooperation level of the child, also play a role. One study reported high disapproval rates; however, when more advanced behavioural techniques such as DGA were avoided by treatment with SDF, the acceptance rates increased [38].

The results reported in this study should be interpreted alongside its limitations. The effectiveness of SDF treatment was evaluated by performing a retrospective analysis. While comparing results from a retrospective study to those of a clinical trial cannot be considered completely valid, the findings from this study represent long-term outcomes and the prognosis of the treated teeth after SDF application. An advantage of such a study is that the patients recruited have been regularly treated and the results are then assessed without pressure on practitioners being aware of participating in a clinical study. In addition, the analysis was carried out under the strict evaluation of the treated teeth using pre-set criteria determining the levels of success, minor or major failures. The surveying of dental professionals included participants employed or part of a post-graduate program at a university paediatric dentistry department; this may pose a concern as the findings cannot be generalised.

Lastly, future efforts should aim to incorporate different remineralising agents in addition to fluorides. A proactive action could be necessary to reduce the incidence of caries in children using products, such as substances based on biomimetic hydroxyapatite, that are showing similar potential in remineralisation [39]. Also, further research is needed to develop a clear treatment plan following SDF treatment. This includes longitudinal studies evaluating the success rates and longevity of direct restorative treatment after SDF application, such as GIC sealants and preformed metal crowns or the further inactivation of caries with multiple SDF applications.

## 5. Conclusions

To the best of our knowledge, this is the first time the use of SDF has been assessed in Germany. The findings of this study give an overview of the treatment of SDF in children and its acceptance by parents and dental practitioners. SDF was found to be effective in treating young children with high caries risk in the long-term, which was perceived as acceptable by dental practitioners and parents equally. We can, therefore, conclude that, with proper diagnosis and sufficient awareness of dentists and parents, SDF can be particularly beneficial for at-risk patients whose treatment could otherwise be very challenging.

## Figures and Tables

**Table 1:** Assessment criteria for the outcome analysis of treated teeth considering the two indications for SDF use.

Success
Dental caries Caries arrested (hardness/softness; lesion feels hard on gentle probing; characteristic SDF discoloration/black staining reported) and no further treatment required. Caries arrested and tooth restored. No clinical signs or symptoms of irreversible pulpal pathology. Tooth exfoliated without minor or major failure. Dentin hypersensitivity (due to MIH) Reported reduced MIH hypersensitivity, tooth not restored. Reported reduced MIH hypersensitivity, tooth then restored.
Minor Failure
Dental caries Caries progression (hardness/softness; soft/leathery lesion on gentle probing; partially achieved/no reported SDF discoloration/black staining). Signs or symptoms of reversible pulpitis treated without requiring pulpotomy or extraction. Dentin hypersensitivity (due to MIH) Hypersensitivity persisted and required other treatment without pulpotomy or extraction.
Major Failure
Signs or symptoms of reversible pulpitis (no spontaneous pain) requiring pulpotomy. Signs or symptoms of irreversible pulpitis (spontaneous/persistent pain) or dental abscess requiring pulpectomy or extraction.

**Table 2:** Demographics and clinical characteristics of the study sample.

Characteristics (*n* = 93)		
Age, mean (SD)		5.3 years (2.9)
Gender, *n* (%)	Female	45 (48.4)
	Male	48 (51.6)
Caries experience, mean (SD)	d_3_mft	6.3 (4.1)
	d_3_t	4.9 (3.9)
	D_3_MFT	2.5 (3.7)
	D_3_T	1.6 (2.8)
Medical history, *n* (%)	Medically unfit	13 (14)
	Medically fit	80 (86)

SD = standard deviation; d = decay; m = missing; f = filled; t = teeth.

**Table 3:** Treatment outcome of SDF-treated teeth due to dental caries only.

Outcomes *n* (%)		Treatments Performed	*n* (%)
Success * 381 (85.2)	No restorative treatment performed 218 (57.2)	Further follow-ups **	218 (48.8)
	Further restorative treatment performed 163 (42.8)	SMART-Hall using PMC	79 (17.6)
		Filling (Composite/Compomer)	50 (11.2)
		SMART-Technique with GIC	22 (4.9)
		Anterior strip crowns	12 (2.7)
Minor Failure	Caries progression/Secondary caries 0 (0)		
Major Failure 66 (14.8)	Irreversible pulpitis/Abscess 66 (100)	Extraction	55 (12.3)
		Pulpotomy	11 (2.5)
Total			447

* Caries arrested; no signs/symptoms of irreversible pulpal pathology. ** Oral hygiene follow-ups involve bacterial plaque disclosing, tooth-brushing training, fluoride use, and application according to age, parental/patient active involvement, motivation, and reinforcement, etc. Glass Ionomer Cement (GIC): Silver Modified Atraumatic Restorative Treatment (SMART). SMART-Hall: after SDF application, teeth are restored using preformed metal crowns (PMC).

**Table 4:** Dentists’ opinions of SDF treatment.

Questionnaire Item		*n* (%)
Find SDF a good alternative to the treatment of early childhood caries.	Agree	20 (74.1%)
	Somewhat agree	3 (11.1%)
	Neither	3 (11.1%)
	Somewhat disagree	1 (3.7%)
	Disagree	0 (0%)
Prefer SDF over other options for the treatment early childhood caries.	Agree	9 (33.3%)
	Somewhat agree	9 (33.3%)
	Neither	8 (29.6%)
	Somewhat disagree	1 (3.7%)
	Disagree	0 (0%)
Use SDF treatment for anxious children with caries.	Agree	10 (37%)
	Somewhat agree	12 (44.4%)
	Neither	3 (11.1%)
	Somewhat disagree Disagree	0 (0%) 2 (7.4%)
Consider SDF before restorative therapy or general anesthesia.	Agree Somewhat agree Neither Somewhat disagree Disagree	21 (77.8%) 5 (18.5%) 0 (0%) 1 (3.7%) 0 (0%)
Confidently use SDF based on the researched evidence for its effectiveness.	Agree Somewhat agree Neither Somewhat disagree Disagree	16 (59.3%) 9 (33.3%) 2 (7.4%) 0 (0%) 0 (0%)
Had an experience with parents rejecting SDF treatment due to aesthetics.	Agree Somewhat agree	1 (3.7%) 9 (33.3%)
	Neither	6 (22.2%)
	Somewhat disagree Disagree	9 (33.3%) 2 (7.4%)

## Data Availability

The data are contained within the article. Further inquiries can be directed to the corresponding author.
